# A nanoporous hydrogel-based model to study chemokine gradient-driven angiogenesis under luminal flow†

**DOI:** 10.1039/d4lc00460d

**Published:** 2024-10-09

**Authors:** Nidhi Mote, Sarah Kubik, William J. Polacheck, Brendon M. Baker, Britta Trappmann

**Affiliations:** a Bioactive Materials Laboratory, Max Planck Institute for Molecular Biomedicine, Röntgenstraße 20, 48149 Münster, Germany; b Joint Department of Biomedical Engineering, University of North Carolina at Chapel Hill and North Carolina State University, Chapel Hill, NC, 27514 USA; c Department of Biomedical Engineering, University of Michigan, 2174 Lurie BME Building, 1101 Beal Avenue, Ann Arbor, MI, 48109 USA; d Department of Chemistry and Chemical Biology, TU Dortmund University, Otto-Hahn-Straße 6, 44227 Dortmund, Germany.

## Abstract

The growth of new blood vessels through angiogenesis is a highly coordinated process, which is initiated by chemokine gradients that activate endothelial cells within a perfused parent vessel to sprout into the surrounding 3D tissue matrix. While both biochemical signals from pro-angiogenic factors, as well as mechanical cues originating from luminal fluid flow that exerts shear stress on the vessel wall, have individually been identified as major regulators of endothelial cell sprouting, it remains unclear whether and how both types of cues synergize. To fill this knowledge gap, here, we created a 3D biomimetic model of chemokine gradient-driven angiogenic sprouting, in which a micromolded tube inside a hydrogel matrix is seeded with endothelial cells and connected to a perfusion system to control fluid flow rates and resulting shear forces on the vessel wall. To allow for the formation of chemokine gradients despite the presence of luminal flow, a nanoporous synthetic hydrogel that supports angiogenesis but limits the interstitial flow proved crucial. Using this system, we find that luminal flow and resulting shear stress is a major regulator of the speed and morphogenesis of angiogenic sprouting, whose action is mediated through changes in vascular permeability.

## Introduction

Angiogenesis, the formation of new blood vessels from pre-existing vasculature, is regulated by many biochemical and mechanical cues from the tissue microenvironment.^1,2^ For example, diffusive gradients of pro-angiogenic signaling molecules, such as vascular endothelial growth factor A (VEGF-A) and sphingosine 1-phosphate (S1P), have been identified as major signals that trigger endothelial cell (EC) sprouting into the surrounding extracellular matrix (ECM) at the onset of capillary extension.^3,4^ In addition to chemokine gradients, sprouting ECs are also exposed to mechanical forces resulting from blood flow through the lumen of the parent vessel, which has been shown to regulate EC function by exerting shear stress on their apical surface, parallel to the vessel wall.^5,6^ Despite the obviously important role of both luminal shear stress and chemokine gradients in EC function, it is not known how both cues synergize to control angiogenic sprouting. This is mainly due to the absence of suitable model systems that enable the control of chemokine gradients in the presence of luminal flow through a parent vessel.

Over the last decade, several *in vitro* models have been developed to study the effect of chemokine gradients on surface-seeded ECs cultured under controlled fluid flow rates.^7,8^ However, these 2D systems lack key structural aspects of angiogenesis, in particular the tubular configuration of native parental blood vessels which are embedded in a 3D matrix. To address this shortcoming, recent advances have developed 3D hydrogel-based microfluidic models, in which sprouting is initiated from a parent vessel inside a 3D hydrogel matrix. While some systems have either integrated chemokine gradients^9,10^ or luminal flow,^11^ a system integrating both cues has not been realized to date. A major challenge lies in the setup of chemokine gradients in the presence of luminal flow, where fluid pressure results in interstitial flow inside the surrounding 3D hydrogel matrix that disrupts gradients of small molecules established by diffusive transport.^12^ The extent of interstitial flow arising from perfusion significantly depends on the structural properties of the hydrogel, such as its pore size.^13^ Hence, the choice of a suitable hydrogel material and its associated porosity is a crucial design consideration for a successful *in vitro* angiogenesis model that supports chemokine gradients despite perfusion.

Here, we developed a 3D biomimetic model using a nanoporous, synthetic hydrogel matrix that enables the study of chemokine-gradient driven EC sprouting from a parent vessel perfused with tunable levels of luminal flow. Using this model, we show that adjusting shear stress over a physiologically relevant range regulates the speed and morphogenesis of EC invasion.

## Results

### Design of a 3D microphysiologic model to study chemokine-driven angiogenic sprouting from a perfused endothelialized parent vessel

**Fig. 1:**
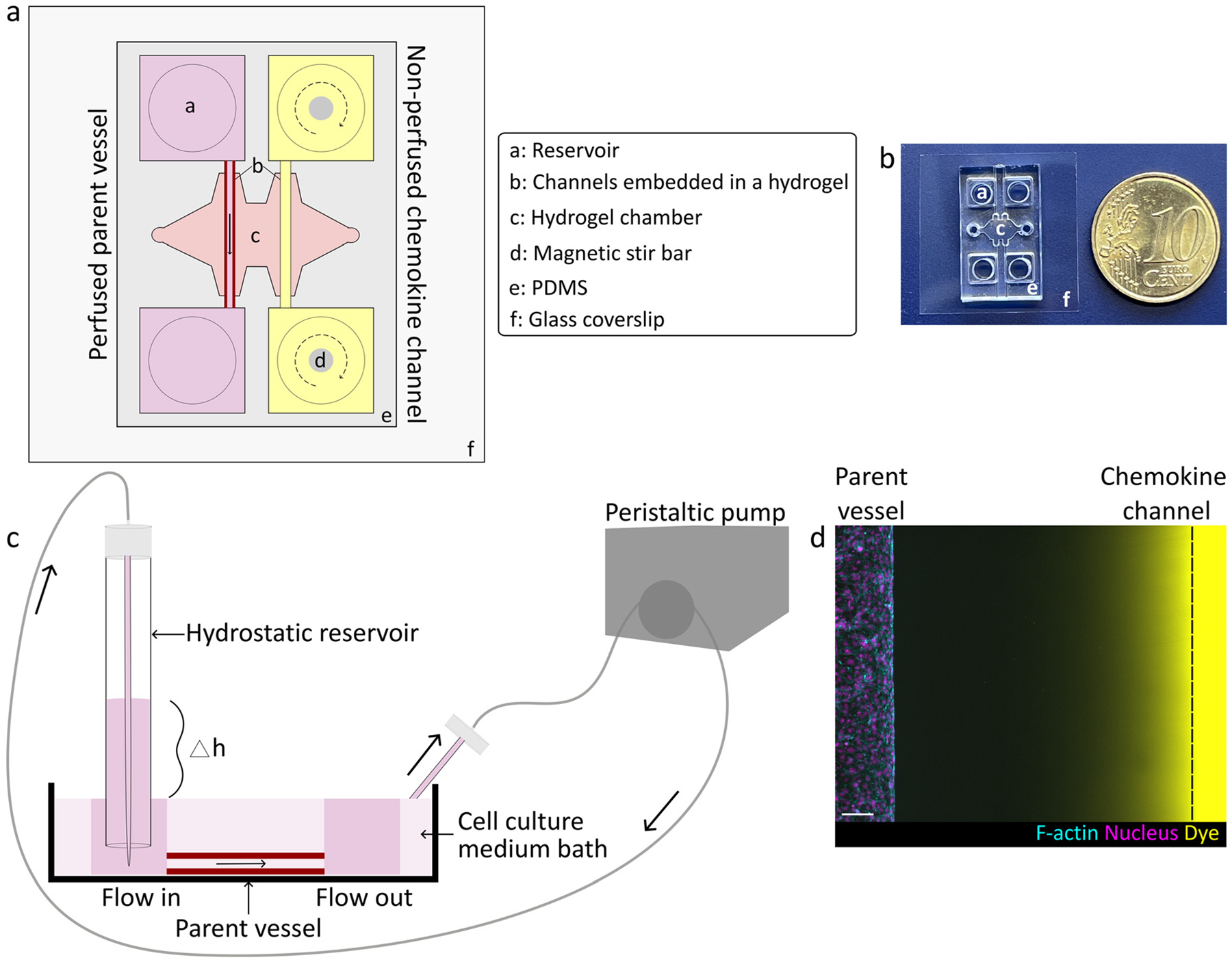
Design of a 3D microphysiologic model to study chemokine-driven angiogenic sprouting from a perfused endothelialized parent vessel. (a) Illustration of PDMS-based microfluidic device consisting of a central hydrogel chamber and two embedded parallel, cylindrical channels. One channel is seeded with ECs serving as a parent vessel, the second, parallel channel is used as a pro-angiogenic chemokine source. (b) Dimensions of the microfluidic device. (c) Schematic of the set-up to apply flow through the lumen of the parent vessel. EC-lined parent vessel is exposed to hydrostatic pressure-driven unidirectional flow established by a difference in medium levels (Δ*h*) between the inlet and outlet. Fluid flow rates are maintained by recirculating the cell culture medium from the surrounding medium bath into the hydrostatic reservoir by a peristaltic pump. Black arrow indicates direction of medium flow. (d) Representative image (maximum intensity projection) demonstrating the set-up of chemokine gradients from the source channel towards the parent vessel. A chemokine mimetic dye (yellow) was used to visualize diffusion inside the hydrogel. ECs are visualized by their cytoskeleton (F-actin: cyan) and nuclei (magenta). Scale bar: 100 μm.

To study how chemokine-guided angiogenic sprouting from a perfused parent vessel is regulated by luminal flow rates, we established a 3D biomimetic model of this process. This model was based on a previously developed microfluidic device that mimics the key structural features of *in vivo* angiogenesis in a controlled, 3D environment.^10^ Specifically, the microfluidic device consisted of two parallel channels fully embedded in a 3D matrix (Fig. 1a and b), such as a collagen type I hydrogel which mimics the stroma of natural tissues and which is known to optimally support the generation of angiogenic sprouts *in vitro*.^9^ One of the channels was seeded with human umbilical vein endothelial cells (HUVECs) that self-assembled into a confluent monolayer, thereby mimicking a parent vessel. To establish controlled fluid flow through the parent vessel, we considered several approaches to perfuse device channels. In the classical approach, EC channels are directly connected to a peristaltic pump to regulate fluid flow; however, this method is known to be technically challenging due to introduction of bubbles that can rupture the channels as well as generation of pulsatile fluid flow, thereby requiring more complicated set-ups including degassers and pulse dampeners.^7,8,11^ To overcome this hurdle, instead of directly connecting a pump to the parent vessel, we introduced a hydrostatic inlet reservoir as an intermediary between the pump and parent vessel, which simultaneously functioned as a buoyancy-based degasser and a pulse dampener. Specifically, we maintained a certain height difference between the inlet reservoir and the outlet to drive unidirectional flow of medium through the lumen of the vessel into a surrounding medium bath, from which the fluid was recirculated into the inlet reservoir by a peristaltic pump (Fig. 1c and S1†). Adjusting the height difference controlled fluid flow rates through the parent vessel over a range of 0.030 to 0.250 mL min^−1^. This setup was amenable to longer-term culture, as tubular endothelial monolayers remained stable for several days (up to three days were tested, but longer culture times are likely possible since EC monolayers showed no signs of defects or rupture). To induce EC sprouting, a pro-angiogenic gradient towards the parent vessel was established inside the hydrogel by addition of a chemokine cocktail (VEGF, S1P and phorbol 12-myristate 13-acetate (PMA)) to the second, parallel channel (chemokine source) (Fig. 1d). In order to avoid depletion of the cocktail ingredients in the non-perfused chemokine channel resulting from diffusion into the hydrogel, the chemokine channel reservoirs were continuously mixed with magnetic stir bars.

### Interstitial flow disrupts chemokine gradients in a microporous, but not in a nanoporous hydrogel

**Fig. 2:**
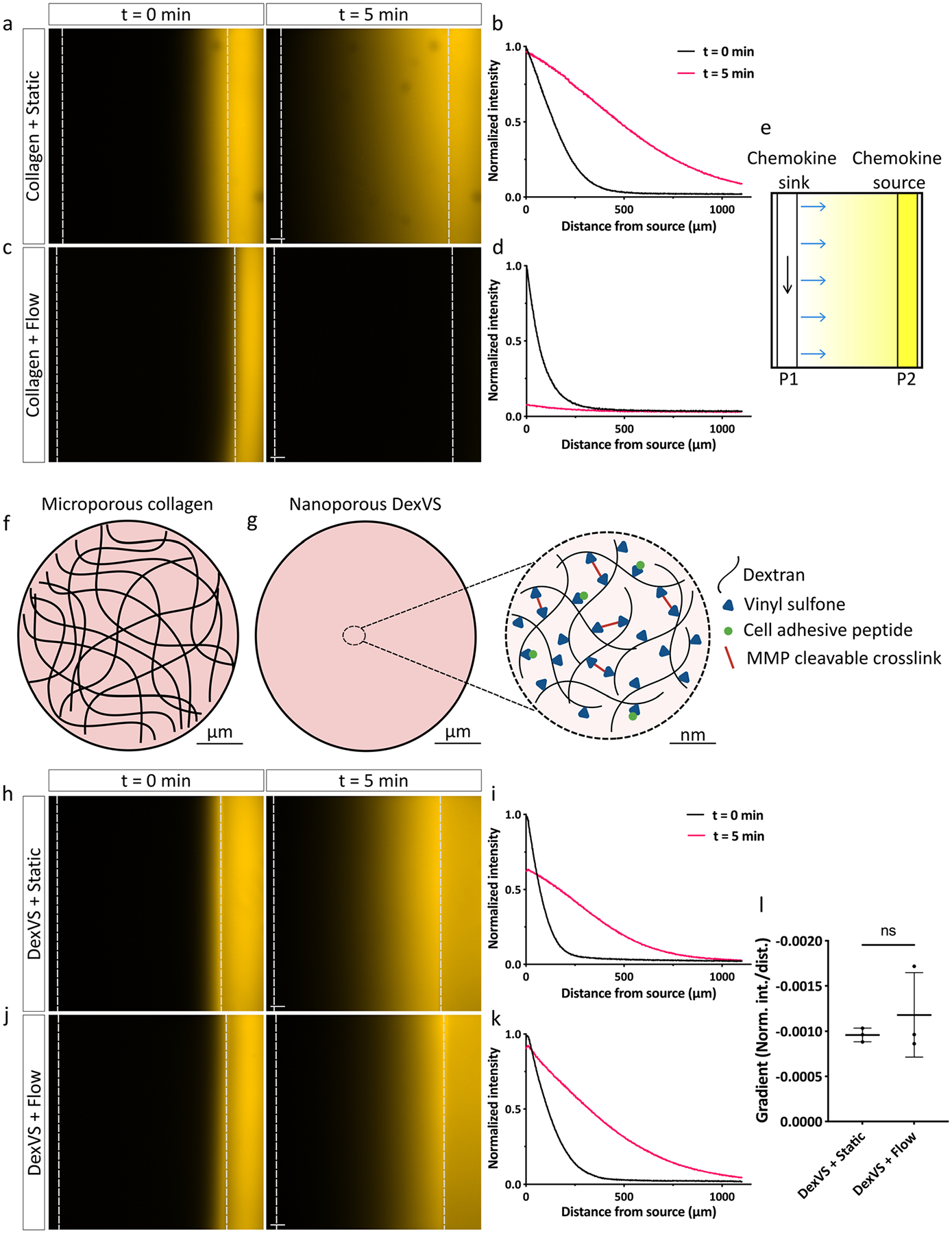
Interstitial flow disrupts chemokine gradients in a microporous, but not in a nanoporous hydrogel. Diffusion of a chemokine mimetic dye (rhodamine B, yellow) through a collagen type I hydrogel, 5 min after dye addition to source channel (right images), compared to 0 min (left images) without (a) and with (c) flow through the sink channel. Shown are fluorescence images of the central channel plane. (b and d) Normalized fluorescence intensity profiles of images in (a and c). (e) Schematic of diffusive gradient set-up from the non-perfused chemokine source and simultaneous convective flow from the parallel perfused chemokine sink through the porous hydrogel due to pressure difference (P1 > P2). Black arrow indicates the direction of luminal flow, blue arrows point in the direction of convection. Illustrations of the structural features of a microporous natural hydrogel based on collagen type I (f) and a nanoporous synthetic DexVS hydrogel shown at the micron- and nano-scale (g). Diffusion of a chemokine mimetic dye (rhodamine B, yellow) through a DexVS hydrogel 5 min after dye addition to the source channel (right images), compared to 0 min (left images) without (h) and with (j) flow through the sink channel. Shown are fluorescence images of the central channel plane. (i and k) Normalized fluorescence intensity profiles of images in (h and j). (l) Quantification of chemokine gradients (normalized intensity/distance (μm)) in DexVS hydrogels without and with flow. Scale bar: 100 μm. *n* = 3 experiments per condition. All data are presented as mean ± s.d., statistical significance was determined from *p* < 0.05 (two-tailed unpaired Student’s *t*-test).

After establishing endothelial parent vessel perfusion, we next tested whether diffusive gradients were able to establish from the static chemokine source channel towards the parallel, perfused parent vessel (chemokine sink), similarly to static, non-perfused conditions previously described in the literature.^9^ To visualize the presence and dynamics of chemokine gradients within collagen type I hydrogels, we monitored the diffusion of the small molecule fluorescent dye rhodamine B, comparable in size to the major pro-angiogenic drivers in our cocktail (S1P and PMA). While in static, non-perfused conditions the rhodamine B gradient was successfully forming over a time span of 5 minutes (Fig. 2a and b), we found that with perfusion through the parent vessel, we could not detect any signal or evidence of a gradient across the matrix spanning device channels following flow initiation. Notably, the dye appeared to be completely washed away from the source channel (Fig. 2c and d). We hypothesized that the introduction of flow through the chemokine sink channel created a positive pressure gradient (P1 > P2) towards the non-perfused chemokine source channel, resulting in interstitial flow across the hydrogel which disrupted the diffusive gradient due to convective transport (Fig. 2e). The build-up of such convective flow could be a direct consequence of the large, micrometer-sized pores of collagen type I hydrogels (Fig. 2f), since analogous observations have been reported for other materials with similar pore sizes before.^14^ Therefore, we speculated that utilizing a nano- instead of microporous hydrogel could overcome the disruption of diffusive gradients due to interstitial flow. Synthetic hydrogels mimicking many characteristics of natural ECMs but composed of a nanoporous polymer network could be ideal matrices. To test this hypothesis, we repeated studies using our previously established dextran-based hydrogel system, which optimally supports the formation of angiogenic sprouts under static conditions.^15,16^ This system is based on a protein- and cell-inert vinyl-sulfone functionalized dextran (DexVS) backbone,^15,17^ to which cysteine-containing cell adhesive peptides (CGRGDS), obtained from natural ECM proteins, can be coupled *via* Michael-type addition. Crosslinking with dicysteine-functionalized matrix metalloproteinase (MMP)-cleavable peptides, derived from the natural cleavage site of collagen type I, allows for proteolytic cell remodeling, which is required for 3D cell migration through the nanoporous hydrogel (Fig. 2g). To test whether chemokine gradients are able to form under luminal flow in DexVS hydrogels, we recorded the diffusion profiles of rhodamine B without and with flow. Indeed, we observed that a gradient of the dye was successfully forming in DexVS hydrogels despite parent vessel perfusion, similar to static conditions (Fig. 2h–k). The steepness of the gradient with and without flow was comparable (Fig. 2l). Importantly, the gradient was maintained over 24 hours in the presence of flow (Fig. S2†). Taken together, these results demonstrate that nanoporous synthetic hydrogels facilitate the establishment of chemokine gradients in 3D hydrogels spanning across two differently perfused channels by suppressing interstitial flow resulting from pressure gradients between them (Fig. S3†).

### ECs in a perfused parent vessel respond to shear stress

**Fig. 3:**
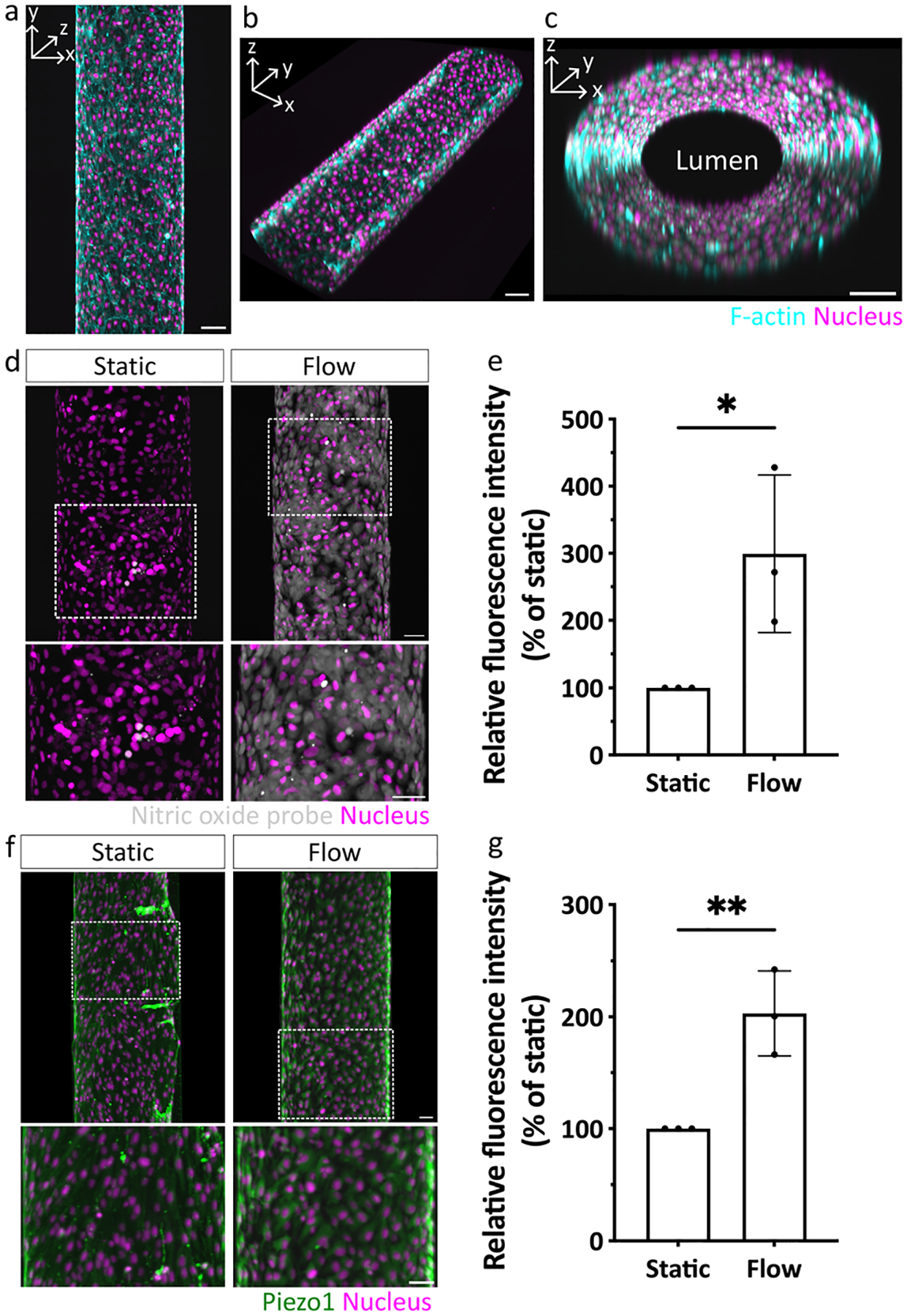
ECs in a perfused parent vessel respond to shear stress. (a–c) Endothelialized channel embedded within a DexVS hydrogel exposed to shear stress of 10 dyn cm^−2^ for 24 h. Shown are representative maximum intensity projections at different angles (F-actin is shown in cyan, nuclei are displayed in magenta). Scale bar: 100 μm. (d) ECs sense flow-induced shear stress, as illustrated by the production of nitric oxide (NO), a shear stress responsive signaling molecule detected by a fluorescent nitric oxide probe, DAF2DA (grey) in parent vessel cultured under flow (right) and static (left) for 1 h. Nuclei are shown in magenta. (e) Quantification of relative fluorescence intensities of DAF2DA in parent vessels cultured under flow, normalized to static conditions. (f) ECs lining the parent vessel show elevated expression of a shear stress mechanosensor, Piezo1 (green), when exposed to flow (right), as compared to static conditions (left) after 72 h, nuclei are shown in magenta. (g) Quantification of relative fluorescence intensity of Piezo1 in parent vessels cultured under flow, normalized to static conditions. Scale bar: 50 μm. *n* = 3 experiments per condition. All data are presented as mean ± s.d., statistical significance was determined from *p* < 0.05 (two-tailed unpaired Student’s *t*-test).

We next investigated whether our DexVS hydrogels also support angiogenic sprouting under flow. In a first set of experiments, we confirmed that seeded ECs were indeed able to form a confluent monolayer in the parent vessel, characterized by vascular endothelial (VE)-cadherin-positive junctions between individual ECs and low permeability (Fig. 3a–c and S4†), similar to in collagen type I hydrogels.^18^ Moreover, the fluorescent bead velocity profile inside the parent vessel confirmed a parabolic flow profile of Poiseuille flow (Fig. S5†) and the actual shear stress calculated from bead velocity was similar to the expected theoretical shear stress value (Table S1†). Next, we tested whether ECs were able to sense and respond to the forces resulting from luminal flow-driven shear stress, which is an important regulator of EC function in general^6^ and during angiogenic sprouting.^19^ Specifically, it has been shown that ECs respond to increased shear stress by upregulating their production of endothelial nitric oxide synthase (eNOS), leading to a rapid rise in intracellular levels of nitric oxide (NO),^20,21^ which in turn has been linked to altered angiogenic sprouting *in vitro*.^7^ To investigate whether ECs in our biomimetic model upregulated NO production under flow, we applied a fluorescent NO probe, 4,5-diaminofluorescein diacetate (DAF2DA).^22^ Indeed, we observed an increase in NO production when ECs were exposed to flow, whereas in static controls, NO was not detected (Fig. 3d and e). In addition to eNOS activity, shear stress is known to activate and upregulate mechanosensitive ion channels, such as Piezo1.^23,24^ We therefore assessed the expression levels of Piezo1 and found it to be significantly higher when ECs were cultured for 72 hours under flow, as compared to static conditions (Fig. 3f and g). These experiments clearly demonstrate that the developed biomimetic model not only supports EC culture in channels under flow, but importantly, recapitulates the cellular response to shear stress *via* activating and upregulating hallmark signaling pathways involved in flow sensing under physiological conditions.

### Shear stress attenuates chemokine-guided angiogenic sprouting in a 3D DexVS hydrogel

**Fig. 4:**
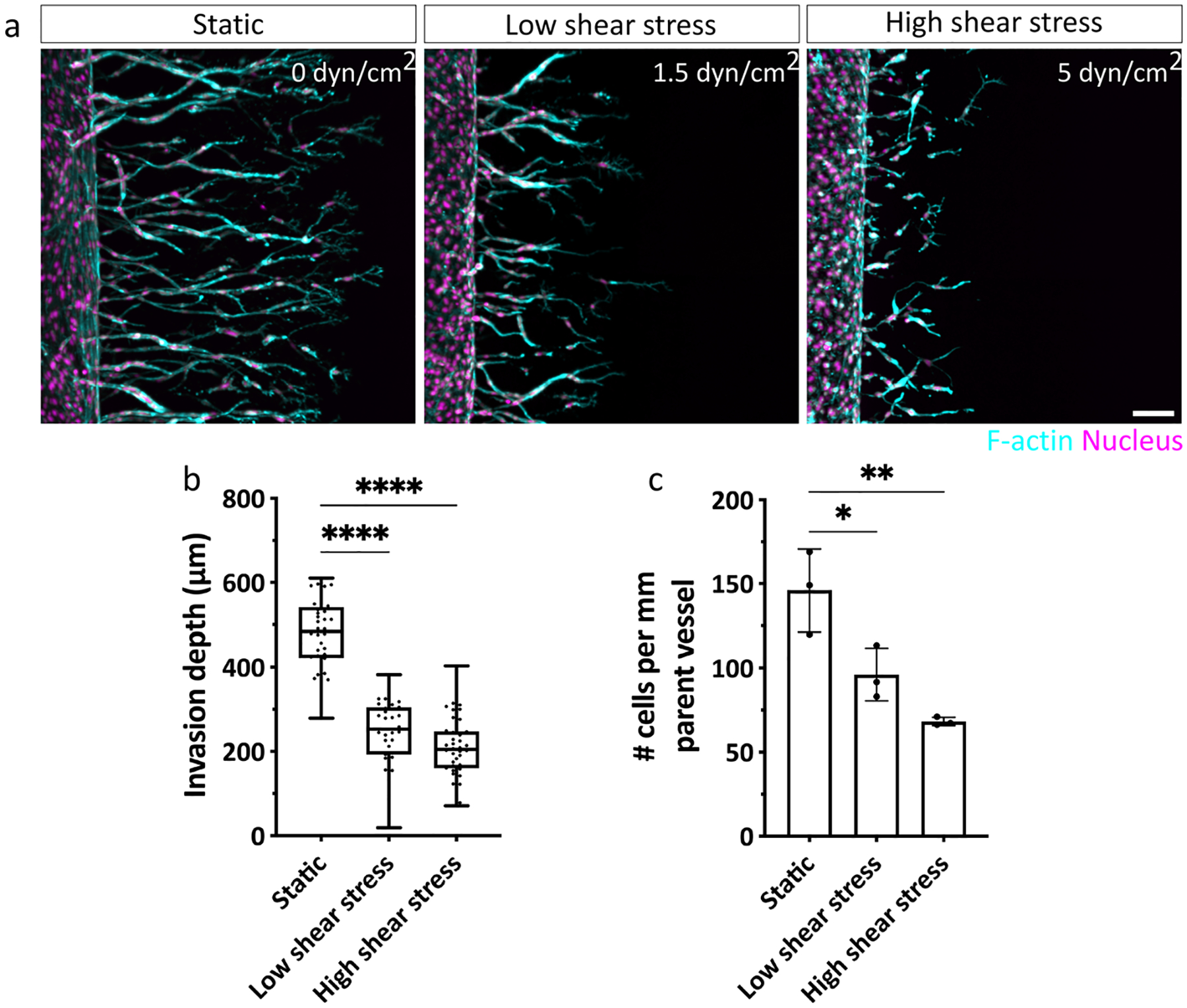
Shear stress attenuates chemokine-guided angiogenic sprouting in a 3D DexVS hydrogel. (a) ECs emanating from parent vessels exposed to no (0 dyn cm^−2^), low (1.5 dyn cm^−2^) or high (5 dyn cm^−2^) shear stress invade DexVS hydrogels at varying speed. F-actin is shown in cyan, nuclei are displayed in magenta. Scale bar: 100 μm. (b) Quantification of invasion depths of angiogenic sprouts after 72 h. *n* = 35–38 sprout segments pooled from 3 independent experiments. (c) Quantification of number of invading cells per mm parent vessel. *n* = 3 experiments per condition. All data are presented as mean ± s.d., statistical significance was determined from *p* < 0.05 (ordinary one-way ANOVA with Tukey’s multiple comparisons test).

With our biomimetic model established and validated for studying chemokine-guided angiogenic sprouting from a perfused parent vessel into a surrounding 3D matrix, we next investigated how ECs respond to different levels of shear stress when migrating along gradients of pro-angiogenic chemokines (Fig. S6†). To do so, we examined EC invasion into 3D DexVS hydrogels in the presence of a range of physiologically relevant shear stress levels cells typically experience in veins (*ca*. 1–6 dyn cm^−2^).^2^ Specifically, we adjusted fluid flow rates through the parent vessel to generate no (0 dyn cm^−2^), low (~1.5 dyn cm^−2^) and high (~5 dyn cm^−2^) vessel wall shear stress and initiated angiogenic sprouting for three consecutive days. While ECs were not triggered to sprout by luminal flow-induced shear stress alone (Fig. S7†), we found that in addition, a pro-angiogenic gradient was required to initiate sprouting (Fig. 4). Interestingly, invasion speeds were diminished in ECs exposed to shear stress (both low and high), compared to static conditions, in which ECs migrated the farthest (Fig. 4a and b). This difference in invasion speed was accompanied by a pronounced decrease in the number of ECs invading DexVS under flow, compared to static conditions (Fig. 4c). Importantly, the observed differences in migration phenotypes were not due to variations in chemokine gradients between static and flow cultures (Fig. 2l and S2c†), but appeared to stem from luminal flow-generated shear forces.

### Shear stress regulates sprout morphogenesis and multicellularity

**Fig. 5:**
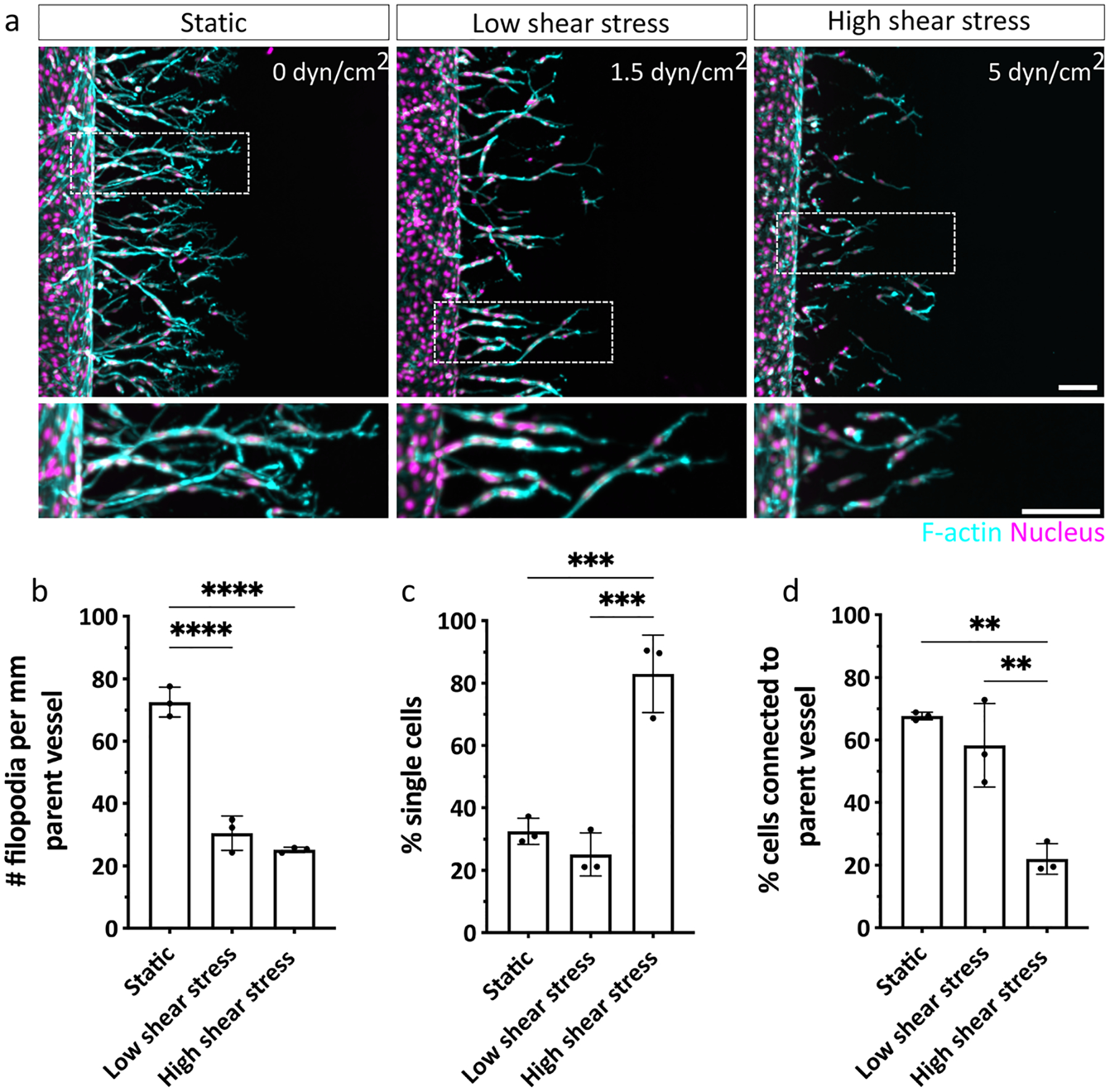
Shear stress regulates sprout morphogenesis and multicellularity. (a) Morphology of ECs emanating from parent vessels exposed to no (0 dyn cm^−2^), low (1.5 dyn cm^−2^) or high (5 dyn cm^−2^) shear stress invading DexVS hydrogels. Samples were fixed at similar invasion depths (static condition fixed after 44 h, high and low shear stress after 72 h of culture). F-actin is shown in cyan, nuclei are displayed in magenta. Scale bar: 100 μm. (b) Quantification of number of filopodia per mm parent vessel. (c) Quantification of percentage cells invaded as single cells relative to total number of cells in the hydrogel. (d) Quantification of percentage cells connected to the parent vessel with respect to total number of invading cells in the hydrogel. *n* = 3 experiments per condition. All data are presented as mean ± s.d., statistical significance was determined from *p* < 0.05 (ordinary one-way ANOVA with Tukey’s multiple comparisons test).

Since we observed differences in the invasion speed and density of ECs, we next aimed to characterize the shear stress-dependent sprouting phenotype in more detail. In particular, we have previously shown that physical properties of the matrix keenly regulate sprout multicellularity,^16^ and thus, we sought to test whether fluid forces similarly controlled this key aspect of sprout morphogenesis. Since ECs exposed to shear stress invaded more slowly, a comparison of sprout phenotypes could be impacted by variations in invasion depths of sprouts. Specifically, different invasion depths result in varying distances of sprouting ECs from the chemokine source channel and hence, variable concentrations of pro-angiogenic factors ECs experience. To exclude this potentially confounding parameter in our analysis, we repeated the sprouting experiments in the absence and presence of flow, but fixed samples at different time points when ECs achieved similar invasion depths (Fig. 5a). Interestingly, we noted shear-stress induced changes in the morphology of the leading tip cells, characterized by narrower branches and a reduced number of filopodial protrusions, compared to static controls in which tip cells displayed highly branched filopodial structures (Fig. 5b). This observation is in line with previous 2D studies showing a shear-stress induced downregulation of ROBO4 (roundabout 4) and CLEC14A (C-type lectin 14A) genes, which are both critical to filopodia formation and EC migration during angiogenesis.^25,26^ Increased shear stress not only altered tip cell morphology, but also significantly impacted sprout progression and multicellularity. While ECs cultured under static or low shear stress conditions primarily migrated as multicellular strands (indicated by low percentage of single cells) that remained connected to the parent vessel, high shear stress caused ECs to migrate as scattered, individual cells (Fig. 5c and d). This observation could suggest that migrating ECs still experience some level of shear forces related to the perfused parent vessel. To test this possibility, we stained for the mechanosensor Piezo1. Indeed, while ECs very close to the parent vessel were still characterized by elevated levels of Piezo1, this effect was reversed in cells that had invaded further into the hydrogel (Fig. S8†). This result demonstrates that ECs inside the matrix no longer sense shear stress, which is in line with our key observation that DexVS hydrogels suppress interstitial fluid flow. Taken together, the sprouting experiments clearly demonstrate that forces from fluid flow not only alter EC signaling in the parent vessel by activating mechanosensitive pathways (which has been shown previously^27^), but also for the first time uncovers a functional role of luminal flow-induced shear stress in the regulation of angiogenic sprout morphogenesis.

### Increased endothelial permeability rescues connectivity of sprouts to parent vessel under high shear stress

**Fig. 6:**
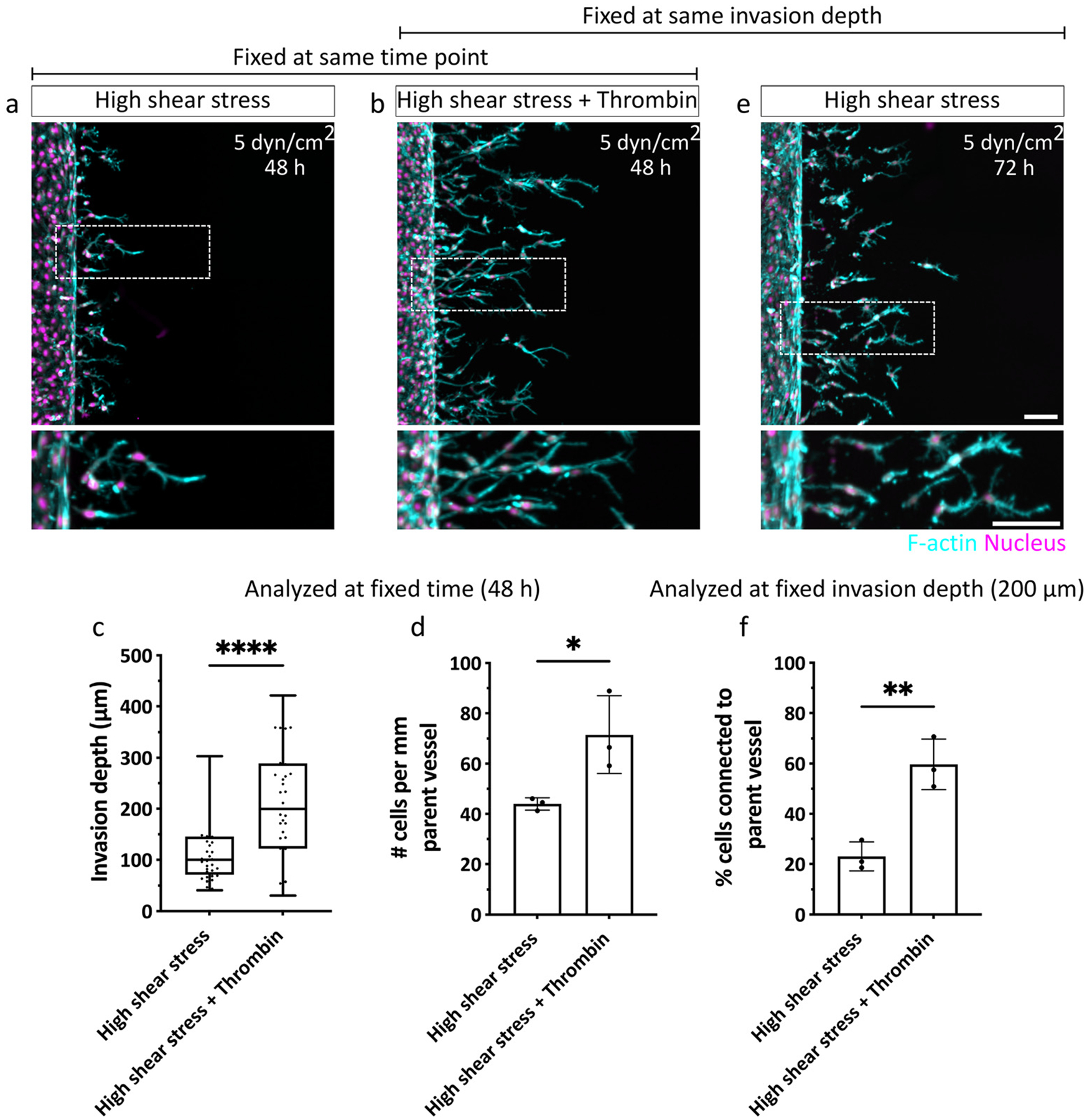
Increased endothelial permeability rescues connectivity of sprouts to parent vessel under high shear stress. (a and b) Parent vessel-lining ECs exposed to high shear stress (5 dyn cm^−2^) invade DexVS hydrogels in the absence and presence of thrombin. Samples were fixed after 48 h of culture. (c) Quantification of invasion depths of angiogenic sprouts after 48 h of culture without and with thrombin. *n* = 36–37 sprout segments pooled from 3 independent experiments. (d) Quantification of number of invading cells per mm parent vessel after 48 h of culture without and with thrombin. *n* = 3 experiments per condition. (e) ECs exposed to high shear stress (5 dyn cm^−2^) invade DexVS hydrogels in the absence of thrombin fixed at similar invasion depth as (b) (high shear stress with thrombin). F-actin is shown in cyan, nuclei are displayed in magenta. Scale bar: 100 μm. (f) Quantification of percentage cells connected to the parent vessel relative to the total number of invaded cells in the hydrogel (in samples fixed at similar invasion depth). *n* = 3 experiments per condition. All data are presented as mean ± s.d., statistical significance was determined from *p* < 0.05 (two-tailed unpaired Student’s *t*-test).

**Fig. 7:**
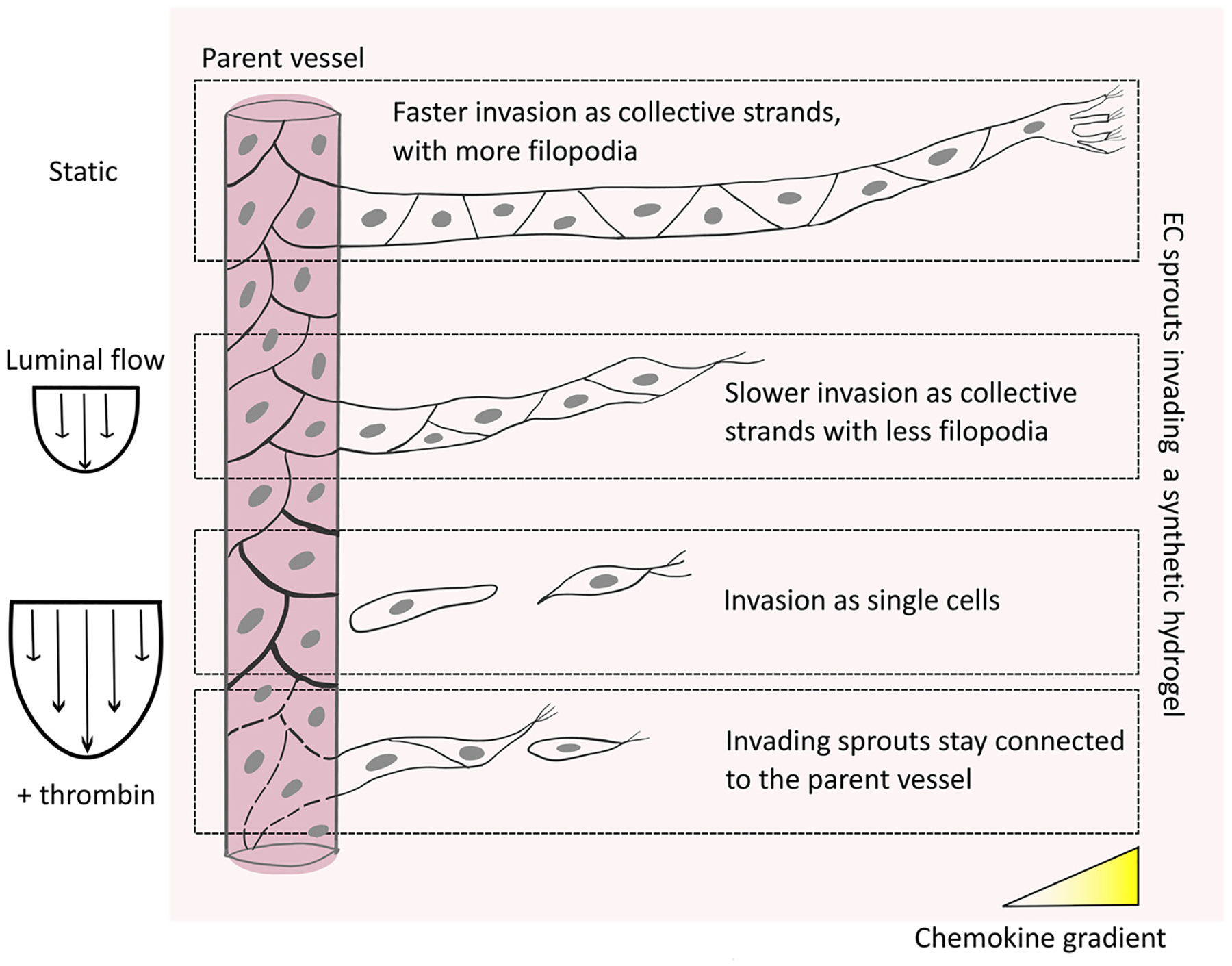
Model of EC response to different levels of shear stress during chemokine-guided angiogenic sprouting. Angiogenic sprouting of ECs from a perfused parent vessel into a 3D matrix as a response to chemokine gradients is regulated by luminal flow-induced shear stress. Under static conditions, ECs primarily migrate as collective strands connected to the parent vessel. Exposing ECs in the parent vessel to low levels of shear stress slows down invasion of cells, accompanied by a phenotypic change of the tip cells, which display a reduced number of filopodial protrusions. Even higher shear stress levels slow down angiogenic sprouting, accompanied by a disconnection of angiogenic strands from the parent vessel. This phenotype is rescued by destabilizing cell–cell junctions and increasing vascular permeability in the parent vessel, which results in a larger fraction of cells staying connected to the parent vessel.

Given the importance of sprout connectivity to the parent vessel for functional angiogenesis,^10^ we next investigated the mechanism underlying the observed shear-stress induced detachment of angiogenic sprouts from the EC channels. The initiation of physiological angiogenesis requires destabilization of the endothelial monolayer in the parent vessel, which enables the escape of leading tip and following stalk cells *via* loosening their cell–cell adhesion with neighboring cells in the parent vessel.^3,28^ It is well established that the shear stress (>3 dyn cm^−2^) generated by steady physiological luminal flow promotes barrier function and decreases permeability of the endothelium by stabilizing and strengthening cell–cell junctions.^18,29^ Thus, we hypothesized that the slow EC invasion observed under high shear stress could result from a decrease in endothelial permeability and tighter EC intercellular junctions, which collectively slow the escape of ECs from the parent vessel. This impaired exiting of invading ECs from the parent vessel, in turn, could mean that stalk cells, which are required to maintain sprout connectivity to the parent vessel, are insufficiently supplied. To test this hypothesis, we increased endothelial permeability under high shear stress by exposing ECs in the vessel to a well-known permeability inducer, thrombin,^30^ which triggers a redistribution of VE-cadherin leading to the formation of intercellular gaps,^31^ and increases permeability of the endothelial monolayer (Fig. S9†). Indeed, when thrombin was added to ECs sprouting for 48 hours under high shear stress, invasion speed was faster and the number of invading cells increased, compared to non-treated controls (Fig. 6a–d). Importantly, when samples were fixed at constant invasion depth (200 μm) to allow for comparable sprout morphogenesis analysis, we found that treatment with thrombin rescued endothelial sprout connectivity to the parent vessel in the presence of flow, demonstrated by VE-cadherin-positive junctional connections (Fig. 6b, e, f and S10a†), and was also comparable to static controls fixed at similar invasion depth (Fig. S10b†). Taken together, our studies suggest that flow-induced changes in vascular permeability regulate the chemokine-stimulated escape of ECs from the parent vessel into the surrounding matrix, which directly impacts sprout morphogenesis. Compared to static conditions, in which ECs invade as multicellular strands that remain connected to the parent vessel, exposure to luminal flow reduces the number and speed of invading cells, which at higher flow rates, results in single-cell migration. The connectivity to the parent vessel under high flow can be rescued by increasing endothelial permeability (Fig. 7).

## Discussion

Given the central role of angiogenesis during tissue homeostasis and disease, many *in vitro* models that recapitulate this complex process within well-controlled and tunable environments have been developed towards gaining deeper mechanistic insights. Traditionally, the migratory response of ECs to pro-angiogenic chemokines has been probed in trans-well assays, in which ECs are seeded on a porous membrane of a cell culture insert and induced to sprout by addition of a chemokine solution into the well below.^32^ While this assay recapitulates the important chemokine-guided initiation step of *in vivo* angiogenesis, it lacks to incorporate forces from luminal blood flow, an important regulator of angiogenesis.^2,33^ In particular, the presence of fluid forces has been shown to regulate the function of cultured ECs by rendering their gene expression profiles significantly more similar to *in vivo* ECs, compared to static cultures, highlighting the need for incorporating vessel perfusion in *in vitro* models that better recapitulate physiologic EC phenotypes.^34^ Therefore, more advanced models have recently sought to mimic angiogenic sprouting by not only exposing ECs to chemokine gradients, but also luminal flow; however, how the resulting shear stress impacts EC invasion is still controversially debated. For example, some studies have reported an inhibitory role of luminal flow in VEGF-induced EC sprouting.^7,8^ However, the microfluidic devices used in these studies are based on surface-cultured ECs, lacking the tubular, 3D geometry of native blood vessels, whose curvature has been demonstrated to impact the EC response to luminal flow.^35^ Other studies have shown that in contrast to cells cultured on flat surfaces, sprouting from a 3D tubular, endothelialized channel is actually enhanced by luminal flow,^11^ further demonstrating the importance of the 3D format of EC vessels in regulating angiogenesis and highlighting the need to recapitulate mechanical and structural aspects consistent with what occurs *in vivo*. To fill this gap, more advanced microfluidic models in which ECs seeded in tubular parent vessels are induced to sprout into a surrounding 3D hydrogel matrix are ideal tools.^9,10^ While some of these models have been developed to include luminal flow, none of them have recapitulated chemokine gradient-driven angiogenic sprouting from perfused 3D parent vessels in the absence of other confounding mechanical forces, in particular interstitial fluid flow resulting from pressure differences between the parent and chemokine source vessels. Here, by making use of nanoporous DexVS hydrogels, we have taken these models an important step further by integrating controlled and tunable flow through the parent vessel without concurrent introduction of interstitial flow, which allows for the first time to study the role of luminal flow through a 3D cylindrical parent vessel in chemokine-guided angiogenic sprouting in the absence of other confounding parameters.

The development of angiogenesis models that incorporate chemokine gradients in a 3D tissue matrix in the presence of luminal flow through a parent vessel critically requires the right choice of a hydrogel material. In this context, it is particularly important to consider that in contrast to static systems, in which chemokines are predominantly disseminated within the porous ECM by diffusive transport,^36^ the introduction of flow gives rise to convective transport through the ECM that impacts chemokine distribution.^19,37^ In addition to the chemical properties of the chemokines, such as hydrophobicity and size, transport of these molecules depends on the structural properties of the surrounding ECM, in particular pore size, fiber diameter and arrangement. For example, diffusive and convective flow both increase with larger ECM pore size.^13^ Importantly, natural hydrogels based on collagen type I or fibrin, both commonly used in microfluidic models of angiogenic sprouting, have large, micrometer-sized pores that engender high levels of convective flow, thereby strongly impacting the diffusion of chemokines.^14^ In these hydrogels, stable chemokine gradients cannot be established under flow conditions, as shown here. To address this challenge, here, we used synthetic nanoporous hydrogels characterized by small, nanometer-sized pores,^38^ which prove relatively impervious to convective interstitial flow emanating from a perfused parent vessel, in turn allowing for the establishment of chemokine gradients. ECs in the parent vessel showed typical responses to chemokine gradients as well as shear stress induced by luminal flow, thereby making this model an ideal system to study how luminal flow specifically regulates chemokine gradient-driven angiogenic sprouting without the interference of interstitial flow.

Using this model, we found that increased fluid flow-induced shear stress lowers EC migration speed. This finding is in line with a large body of literature that demonstrates the importance of shear stress in the maintenance of endothelial barrier integrity, mainly through stabilization of adherens junctions between ECs.^29^ As a consequence of barrier tightening under flow, here, we show that ECs exit the parent vessel more slowly and, due to the lack of follower cells, also more single-cellularly. Indeed, destabilizing EC junctions in the vessel by a known permeability inducer, thrombin, restored the levels of migration speed, density of exiting cells and connectivity of cells to the parent vessel. Our finding of decreased EC exiting under flow is also in line with *in vivo* work that has established physiologically high levels of blood flow as atheroprotective, keeping ECs in a quiescent state and suppressing angiogenesis.^39^ Apart from regulating permeability by acting on cell–cell junctions, shear stress activates many known flow-sensitive mechanosensors^21^ present within ECs, whose conversion of physical cues into biochemicals signals through cellular mechanotransduction events has been studied in great detail.^40^ While not much is known about the functional consequences of flow-induced EC activation for angiogenesis, we have recently shown that mechanical properties from the ECM regulate the multicellularity of angiogenic sprouts.^41^ Specifically, in highly crosslinked, stiff matrices that are characterized by a high resistance towards cellular proteolytic cleavage, ECs switch to an actomyosin contractility-based single-cell migration mode. This migration strategy is similar to the actomyosin-dependent lobopodial migration cancer cells and fibroblasts adopt in highly confined environments in order to physically push through the matrix.^42,43^ Here, we have shown for the first time that the collectivity of chemokine-guided angiogenic sprouts is regulated by fluid forces through the parent vessel, however, the full molecular mechanism underlying single cell migration remains to be discovered. Given the importance of other mechanical signals, in particular from the ECM, it will be necessary to elucidate the interplay between luminal flow and other currently unexplored mechanical signals originating from the tissue microenvironment of blood vessels in the future, for which our microfluidic model is ideally suited since it is based on a synthetic hydrogel with tunable mechanical properties. Currently, our developed tool is limited by ECs being the sole cellular component, and future studies will have to incorporate other cell types that regulate blood vessel development and homeostasis, such as pericytes and smooth muscle cells.

In addition to providing an improved understanding of the regulatory role of luminal flow in chemokine-guided angiogenesis, our model will also be directly applicable to angiogenesis in disease states that have been linked to abnormal blood flow patterns. For example, during vascular malformations, blood flow is disturbed; if and how this impacts angiogenesis is not at all known, mainly due to the complexity of the disease. Here, our model can help elucidate molecular mechanisms that can then be probed *in vivo* in follow-up studies. While the current setup of our model is based on a generic source of ECs as well as cell-adhesive and MMP-cleavable matrix molecules, we believe that this approach will be extendable to mimic organotypic vascular niches under flow by incorporation of tissue-specific (peri-) vascular cell types and ECM molecules, *e.g*. to mimic the blood–brain barrier. In addition to studying angiogenesis, the developed model will be applicable to many other biological processes that depend on chemokine gradients, such as cancer metastasis or leukocyte trans-endothelial migration during inflammation.

## Materials and methods

### Reagents

All reagents were purchased from Sigma-Aldrich, unless indicated otherwise.

### Adhesive and MMP cleavable peptides

The cell-adhesive ligand CGRGDS (RGD) and matrix metalloproteinase (MMP)-cleavable crosslinker peptide of native collagen degradability KCGPQGIAGQCK (NCD) were custom synthesized (provided as HCl salt) by Genscript at >95% purity.

### Microfluidic device fabrication

Microfluidic devices were fabricated as previously described.^10^ In short, 3D printed molds were designed in AutoCAD and printed *via* stereolithography by Protolabs, USA. The mold was screwed to a glass plate (Rettberg) and polydimethylsiloxane (PDMS) (10 : 1 base : curing agent ratio) was replica casted, resulting in a set of eight microfluidic devices which were cut into individual devices. Circular reservoirs (3 mm diameter) were punched with biopsy punches (SMI) to allow connection of the hydrostatic reservoir required for experiments under flow. To prevent dilution of the pro-angiogenic cocktail added to the chemokine source channel, through the surrounding EGM-2 bath, circular PDMS walls (4 mm diameter) were attached on top of the device. The device was cleaned with sticky tape, plasma-treated (Femto, Diener electronic), and bonded to plasma-etched glass coverslip, followed by UV-sterilization.

### Preparation of natural, collagen type I hydrogels inside the microfluidic device

To promote attachment of collagen to PDMS, the central gel chamber of the microfluidic device was functionalized with an aqueous 0.1% (w/v) poly-L-lysine followed by 1% (w/v) glutaraldehyde solution. The device was washed overnight with water, treated with 70% ethanol and air dried to remove excess water and ethanol. To form cylindrical channels inside the hydrogel, two acupuncture needles (Hwato, 300 μm diameter) were coated with a 0.4% aqueous solution of bovine serum albumin (BSA) and inserted into the device through the needle guides. A 2.5 mg mL^−1^ collagen type I (rat tail, Corning, 3.28 mg mL^−1^ stock) solution was prepared in M199 medium containing 10 mM HEPES and 0.035% (w/v) NaHCO_3_ on ice, the pH was adjusted to 7.0 and the precursor solution added into the central gel chamber. The solution was allowed to polymerize at 37 °C for 30 min in a humidified dish (using wet tissues) sealed with parafilm, without exposure to air. After polymerization, PBS was added to the device and incubated overnight, followed by needle extraction and thorough washing with PBS on a rocker at 37 °C.

### Synthesis of vinyl sulfone-functionalized dextran (DexVS)

Dextran was functionalized with vinyl sulfone groups following a previously established protocol.^44^ Briefly, divinyl sulfone (2.48 mL) was added dropwise to dextran (2.0 g, MP Biomedicals, MW 86 000 Da) dissolved in an aqueous sodium hydroxide solution (0.1 M, 200 mL) at room temperature under vigorous stirring. The reaction was stopped after 5 min by adjusting the pH to 5 with hydrochloric acid solution (2.4 M). The product was purified by dialysis (SnakeSkin^™^ Dialysis Tubing, Life Technologies, 10 000 Da MW cutoff) against Milli-Q water for 3 days, with two water exchanges daily. The dialyzed product was lyophilized and analyzed by ^1^H-NMR spectroscopy. The final product with a vinyl sulfone/dextran repeat unit ratio of 0.5 was obtained.

### Preparation of synthetic, MMP cleavable DexVS hydrogels inside the microfluidic device

DexVS and RGD were dissolved in phenol red – PBS under sterile conditions, aliquoted and stored at −80 °C till further use. A solution of DexVS (final concentration of 4.2% w/v) and RGD (final concentration of 6 × 10^−3^ M) was prepared on ice. The coupling of RGD to DexVS was initiated *via* Michael-type addition reaction by adjusting the pH to ~7.5 using NaOH (1 M), followed by 30 min incubation at room temperature. To form tubular channels, two acupuncture needles (Hwato, 300 μm diameter) were coated with a 5% (w/v) aqueous gelatin solution and cooled to 4 °C before inserting them into the device. The hydrogel precursor solution was cooled again on ice and NCD peptide (26.4 × 10^−3^ M) was added. The hydrogel crosslinking was initiated by adjusting the pH to ~7.5 using NaOH (0.2 M) and the solution was immediately added to the central gel chamber of the microfluidic device. The sample was incubated in a dish at room temperature for 30 min to ensure full gelation in a humidified environment (by covering the outside of the dish with wet tissues). The polymerized hydrogel was hydrated in PBS, followed by overnight incubation at 37 °C before needle extraction. DexVS hydrogels used in this study had a Young’s modulus of 1.05 kPa, as characterized using a nanoindenter (Piuma, Optics 11 V3.4.7, Netherlands).

### Cell culture and seeding in the microfluidic device

HUVECs were purchased from Lonza (catalog number C2519A) and cultured in fully supplemented endothelial cell growth medium 2 (EGM-2) (PromoCell) containing additional 250 ng mL^−1^ amphotericin B and 10 μg mL^−1^ gentamicin (Gibco). Cell cultures were maintained in incubators with constant humidity at 37 °C and 5% CO_2_. In all assays, HUVECs of passage 4–5 were used. Before seeding, the needle guides were sealed with vacuum grease (Dow Corning) to prevent leakage and the channels were subsequently washed with PBS and EGM-2. HUVECs were briefly trypsinized and a solution of 10 million cells per mL in EGM-2 was prepared. The cell suspension was added to one reservoir of the parent vessel and cells were allowed to attach to the bottom side of the channel for 20 min, followed by seeding of the top channel side (through inversion of the device) with fresh cell suspension for another 20 min at 37 °C. The reservoirs were scratched and unattached cells were washed out of the parent vessel and the device was incubated at 37 °C for 5 h on a rocker at 15° tilt angle (BenchRocker BR2000).

### Application of luminal flow through the parent vessel

To ensure a leak-free device under flow and to limit interstitial pressure gradients, the gel injection ports were sealed with vaseline, lanolin and paraffin (VALAP) wax^18^ and the outlet of the parent vessel was cut open to allow outflow of EGM-2 into the surrounding medium bath. To expose the parent vessel to different magnitudes of shear stress, the required flow rate was calculated based on the following formula:

$$Q=\frac{\pi R^{3}}{4\mu }\tau_{w}$$

where *Q* = volumetric flow rate

*R* = radius of the parent vessel (measured before the start of each experiment)

*μ* = fluid viscosity (Newtonian fluid assumed)

*τ*_w_ = wall shear stress.

A 1 mL pipette tip serving as a hydrostatic reservoir was inserted into the inlet of the parent vessel, and the device was placed in an EGM-2 bath. The hydrostatic reservoir was connected to a peristaltic pump (ISMATEC, MasterFlex) *via* Pharmed BPT tubings (Masterflex, 0.51 mm ID) which recirculated the medium from the bath into the hydrostatic reservoir at the calculated flow rate, thereby maintaining constant exposure to shear stress. During recirculation of medium through the parent vessel, the medium was released into the bath, thereby automatically maintaining the level of medium in the hydrostatic reservoir and the bath. Furthermore, to compensate for the loss of water due to evaporation, the medium in the bath was replenished daily. The chemokine source channel was maintained under static condition and was not perfused.

The device was covered with a PDMS cap having biopsy-punched 7 mm and 4 mm circular holes over the reservoirs of parent vessel and chemokine source channel, respectively, to insert the hydrostatic reservoir and allow daily cocktail exchange during the experiment. The entire setup was then transferred to a cell culture incubator. For repeated use, the tubings were extensively washed with an in-house-prepared ultrasonic cleaning solution containing pentasodium triphosphate, sodium bicarbonate and sodium lauryl sulfate and sterilized by autoclaving or gamma irradiation after each use.

### Angiogenesis assay

To induce angiogenic sprouting, a pro-angiogenic cocktail was added to the chemokine source channel with or without flow in the parallel parent vessel. This cocktail consisted of rhVEGF 165 (R&D Systems, 75 ng mL^−1^, MW 42 000 g mol^−1^), PMA (150 ng mL^−1^, MW 616.83 g mol^−1^), and S1P (Cayman Chemical, 250 nM, MW 379.47 g mol^−1^) in EGM-2. To ensure mixing and homogenous supply of the cocktail ingredients throughout the channel, a magnetic stirring bead (Cowie) was positioned in the chemokine source channel and the device was kept on a magnetic stirrer (Roth) for the entire duration of the assay (44–72 h). For experiments with thrombin, 1 U mL^−1^ thrombin was added to the EGM-2 bath. To test the effect of flow on angiogenic sprouting in the absence of a chemokine gradient, the parent vessel was exposed to a shear stress of 5 dyn cm^−2^ for 72 h. The cocktail and EGM-2 were exchanged daily throughout the course of the experiment.

### Synthesis of FITC-conjugated VEGF-A

Human VEGF-A_165_ was conjugated with fluorescein isothiocyanate (FITC) using FluoReporter FITC Protein Labeling kit (Invitrogen). Briefly, recombinant human VEGF-A_165_ (Peprotech) was added to reactive FITC dye at a ratio of 1 : 100 in sodium phosphate buffer (0.1 M, pH 7.4). The reaction was performed at room temperature in the dark under constant stirring, followed by purification through size-exclusion chromatography using spin-columns provided by the labeling kit. Assuming 85% recovery of labeled-protein, a degree of labeling of 0.5 was estimated. The labeled VEGF was stored in 1% BSA solution at −20 °C.

### Characterization of gradient formation

To visualize the set-up of chemokine gradient inside the hydrogel, the small molecule, chemokine-mimetic dye rhodamine B (10 μg mL^−1^, MW 479.02 g mol^−1^) was added to the chemokine source channel just before the start of the experiment. Time-lapse imaging was performed using the fluorescence microscope Axiovert 200M with built-in ZEN 2 software (blue edition, 2.0.0.0) to monitor the diffusion of rhodamine B for 5 min following addition of the dye, in the absence and presence of luminal flow through the parallel channel. The intensity of rhodamine B that had diffused from the source channel through the hydrogel towards the sink was quantified in ImageJ (1.54 h) using the Plot Profile tool. Intensity measurements were averaged from three different locations per image and normalized to the highest intensity value for the respective *t* = 0 min time-point. To characterize the gradient maintenance over 24 h, rhodamine B (10 μg mL^−1^) and FITC-labeled VEGF-A (10 μg mL^−1^) were added to the chemokine source channel and the gradient was imaged after 24 h. To calculate the steepness of the gradient, the intensity *versus* distance graph was fitted to a simple linear regression model and the slopes were compared between static and flow conditions in DexVS hydrogels.

### Measurement of diffusion coefficients and convective fluid flow in collagen and DexVS hydrogels

To characterize diffusion and convection in collagen and DexVS, fluorescence recovery after photobleaching (FRAP) was performed using a point scanning confocal microscope (Zeiss LSM 880 with built-in Zen software version Black 2.3). Collagen and DexVS hydrogels were incubated with FITC-conjugated VEGF-A (10 μg mL^−1^) overnight. To measure the diffusion coefficient, a spot in the hydrogel region spanning the two non-perfused channels was irradiated with a 488 nm laser to locally bleach the fluorescence. The fluorescence recovery was imaged every second. A VEGF diffusion coefficient of 2.60 μm^2^ s^−1^ was determined for collagen, and of 0.45 μm^2^ s^−1^ for DexVS hydrogels. To visualize the convective fluid flow in the hydrogel resulting from pressure differences between the two channels, the inlet of one channel was connected to a hydrostatic reservoir to introduce luminal flow, and the parallel channel was maintained statically. A hydrogel region near the perfused (positive pressure) channel was photobleached by irradiation with 488 nm laser and the drifting of the spot was monitored by imaging the fluorescence signal every second. For quantification, the center of mass of the spot was calculated in ImageJ and its movement with reference to the channel edge was tracked. The fluid velocity was calculated to be 1.02 μm s^−1^ for collagen and 0.02 μm s^−1^ for DexVS hydrogels.

### Detection of nitric oxide

The shear stress-dependent production of NO by cells in the parent vessel was tested using DAF2DA, a cell membrane-permeable fluorescent probe of NO. Specifically, the parent vessel was exposed to luminal flow for 1 h in the presence of DAF2DA (10 μM) and Hoechst 33342 (0.5 μg mL^−1^, Life Technologies) in the EGM-2 bath, or under static conditions at 37 °C. Images of live cells in the parent vessel were acquired.

### Fluorescent staining, microscopy and image analysis

Upon completion of the sprouting experiment, samples were fixed with 4% paraformaldehyde in PBS with calcium and magnesium (Thermo Fisher Scientific) for 1 h at room temperature, followed by permeabilization with 0.5% Triton X100 in PBS for 1 h at room temperature. To assess Piezo1 expression, samples were blocked with 3% bovine serum albumin for 1 h, incubated with Piezo1 monoclonal antibody (1 : 50, Thermo Fisher Scientific) at room temperature for 1 h, followed by secondary antibody donkey anti-mouse 647 (1 : 1000, Invitrogen), Alexa Fluor 488 phalloidin (1 : 500, for F-actin, Thermo Fisher Scientific) and Hoechst 33342 (1 : 200, for nuclei) for 3 h at room temperature. Devices were washed with PBS-Tween20 overnight at 4 °C after each antibody incubation step. For visualization of angiogenic sprouts, samples were permeabilized and stained with Alexa Fluor 488 Phalloidin and Hoechst 33342 overnight at 4 °C. To visualize cell–cell junctions, samples were stained with Alexa Fluor 647 conjugated CD144 (VE-cadherin) antibody (1 : 100, BD Pharmingen) overnight at 4 °C. Fluorescent images were captured using a spinning disc confocal microscope (Dragonfly by Andor with built-in software Fusion version 2.0.0.13).

For DAF2DA analysis, the sum fluorescence intensity was quantified using IMARIS (version 10.0.1) and background intensity was subtracted. Piezo1 expression in the parent vessel was quantified by manually reconstructing the vessel surface in IMARIS and measuring the sum fluorescence intensity at a constant threshold and the background intensity was subtracted. The intensity of each flow sample was normalized to the corresponding intensity in the static control to compensate for variation in absolute fluorescence intensity between experiments. For better visualization and representation of the parent vessel showing Piezo1 expression (Fig. 3f), EC sprouts from the vessel were masked in IMARIS. All analyses of sprouts were performed manually using ImageJ. Invasion depth was quantified by segmenting maximum intensity projection images at 100 μm intervals and measuring the distance from the parent vessel edge to the nucleus of the farthest cell in each segment. The number of ECs invading the hydrogel in each confocal *z*-stack was determined and divided by the length of the parent vessel. To characterize sprout morphogenesis, filopodia of tip cells directed towards the chemokine gradient with acute angles were counted and normalized to the length of the parent vessel using maximum intensity projection images. Percentage of single cells was quantified as number of individually invaded cells in the hydrogel divided by the total number of invaded cells within each *z*-stack. The percentage of cells connected to the parent vessel was quantified as total number of cells showing F-actin connections to the parent vessel divided by the total number of invaded cells in each *z*-stack.

### FITC dextran leakage assay

A HUVEC-seeded channel was exposed to 5 dyn cm^−2^ shear stress for 24 h. To increase endothelial monolayer permeability, the parent vessel was treated with 1 U mL^−1^ thrombin for 30 min. Then, 70 kDa FITC-dextran (250 μg mL^−1^) solution in EGM-2 (with or without thrombin) was added to the parent vessel and images were acquired at different time points using a spinning disc confocal microscope, in which the sample was maintained at 37 °C and 5% CO_2_. The intensity of FITC-dextran in the hydrogel surrounding the parent vessel was quantified using the Plot Profile tool of ImageJ, and it was normalized to the highest intensity measured.

### Bead tracking for flow characterization

The parent vessel was seeded with lentivirally transduced HUVECs expressing LifeAct-mRFPruby. Flow was initiated inside the parent vessel to expose cells to high shear stress (5 dyn cm^−2^) in the presence of 4 μm diameter fluorescent beads (Invitrogen). Time lapse imaging was performed using the fluorescence microscope Axiovert 200M and images were acquired every 20 ms, with an exposure time of 10 ms, for a time span of 40 s at 10× magnification. Timelapse images of beads were initially processed in ImageJ to adjust contrast. A composite image consisting of an average projection for all time points was generated, and the average image and images at each time point were exported into MATLAB. In MATLAB, a crop rectangle was defined by manually selecting the coordinates of the top and bottom corners of the vessel. For each timepoint, the average image was subtracted to remove any stationary objects from the frame. To remove any gaps in pixels for each streak, a Gaussian filter was applied (*σ* = 1), followed by a successive dilation and erosion. A binary image was created by adjusting the contrast, top-hat filtering, and thresholding. Individual streaks were identified using connected components. Identified objects that were too small (<250 pixels), too large (>1000 pixels), or intersected with the edge of the frame were removed from the data. The remaining streaks were analyzed for major axis length (streak length) and centroid (position in the channel), which values were then converted from pixels to mm. Streak length was converted to bead velocity by dividing by the streak length by the exposure time. The position in the channel and bead velocity data were fitted to a quadratic with the equation:

$$v_{z}(x)=\frac{2Q}{\pi R^{2}}\left(1-{\left(\frac{x}{R}\right)}^{2}\right)$$

where *v*_*z*_ is magnitude of the axial velocity vector, *x* is the distance from the center of the vessel, and *R* is the radius of the channel. The magnitude of *Q* is the volumetric flow rate, which could then be used to calculate wall shear stress *τ*_w_, assuming a Newtonian fluid, using the equation:

$$\tau_{w}=Q\times \frac{4\mu }{\pi R^{3}}$$

where *μ* is the fluid viscosity, assumed to be 10^−3^ Pa s.

### Statistical analysis

Statistical analysis was performed in GraphPad Prism Version 10.1.0. Statistical significance was determined by two-tailed unpaired Student’s *t*-test and ordinary one-way ANOVA with Tukey’s multiple comparisons test. *P* values < 0.05 were considered statistically significant. All data are presented as mean ± standard deviation. Each study was independently replicated three times.

### Software

All schematics were drawn in Inkscape (1.3.2). Illustration for Fig. 7 was hand drawn in Procreate (5.3.7).

## Supplementary Material

Supplemental information

## Data Availability

The data that support the findings of this study are available from the corresponding author upon reasonable request.

## Footnotes

† Electronic supplementary information (ESI) available. See DOI: https://doi.org/10.1039/d4lc00460d
