# Supplementary material for: A nanoporous hydrogel-based model to study chemokine gradient-driven angiogenesis under luminal flow
Source: Lab Chip. Author manuscript; Available in PMC 2026 Jun 16. (PMC13271289; doi:10.1039/d4lc00460d)
Supplement: Supplemental information [file NIHMS2182074-supplement-Supplemental_information.pdf]

Supplementary Information (SI) for Lab on a Chip.
This journal is © The Royal Society of Chemistry 2024

Supplementary Information

A nanoporous hydrogel-based model to study chemokine gradient-driven angiogenesis under luminal flow

Nidhi Mote, Sarah Kubik, William J. Polacheck, Brendon M. Baker, Britta Trappmann*

* Correspondence

Prof. Dr. Britta Trappmann

Tel.: +49 231-755 6746

britta.trappmann@tu-dortmund.de

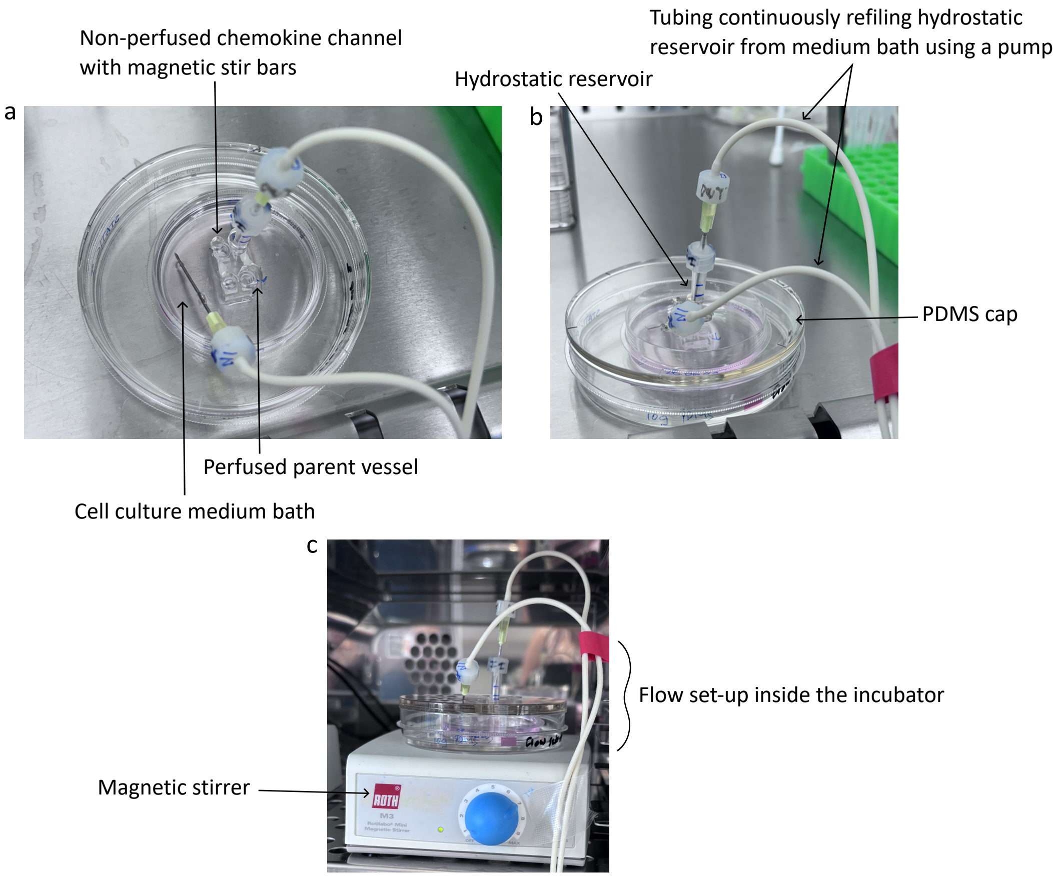

Fig. S1 Illustration of the flow set-up. Top view (a) and side view (b). (c) The set-up is kept on a magnetic stirrer inside a cell culture incubator.

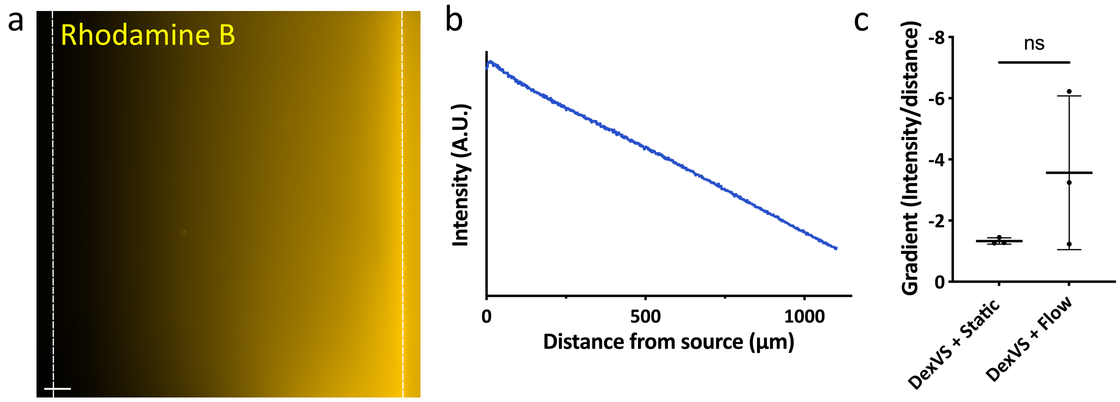

Fig. S2 Maintenance of rhodamine B gradient in DexVS hydrogel over 24 h in the presence of luminal flow. (a) Rhodamine B (yellow) was added to the chemokine source channel and allowed to diffuse through the DexVS hydrogel towards the chemokine sink (parent) vessel under continuous flow. Image was taken after 24 h. (b) Quantification of gradient formation of rhodamine B at 24 h. (c) Quantification of chemokine gradients in DexVS with and without flow at 24 h. Scale bar: 100 µm.

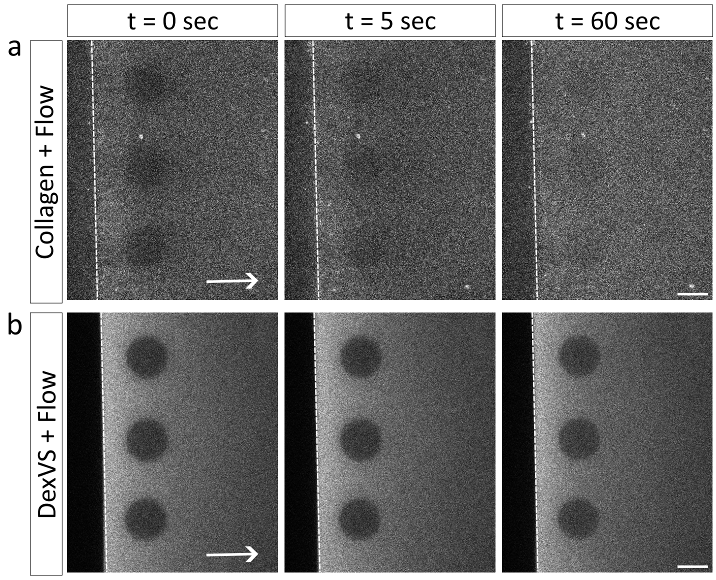

Fig. S3 Visualization of convective fluid flow in collagen and DexVS hydrogels. FRAP experiments were performed to determine the velocity of convective fluid flow through type I collagen and DexVS hydrogels, which was initiated from channel with high (left) to no (right) flow. Fluid velocities were calculated to be 1.02 µm/s for collagen (a) and 0.02 µm/s for DexVS hydrogels (b). White arrows indicate direction of convective flow. Scale bar: 50 µm.

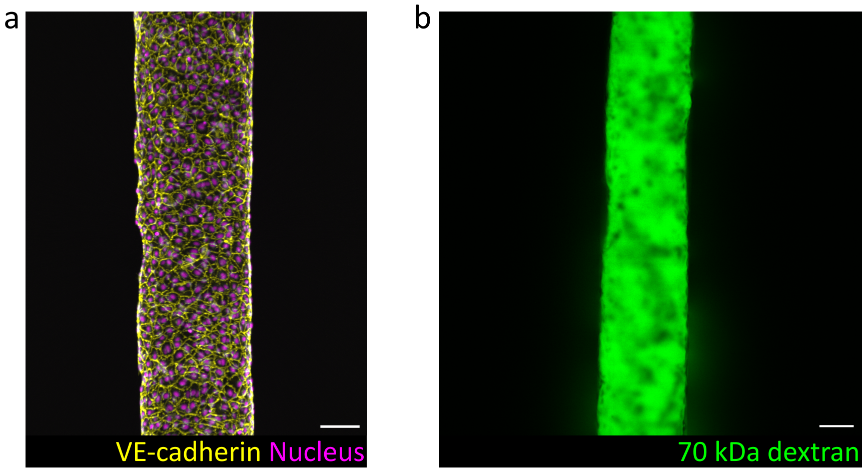

Fig. S4 Integrity and barrier formation of EC-lining parent vessel cultured under flow. (a) ECs form a confluent monolayer and display VE-cadherin positive cell-cell junctions when exposed to flow for 72 h (VE-cadherin is shown in yellow and nuclei are displayed in magenta). (b) Representative image of leakage of 70 kDa dextran, 5 min after injection to the parent vessel which had been exposed to flow for 24 h. Scale bar: 100 µm.

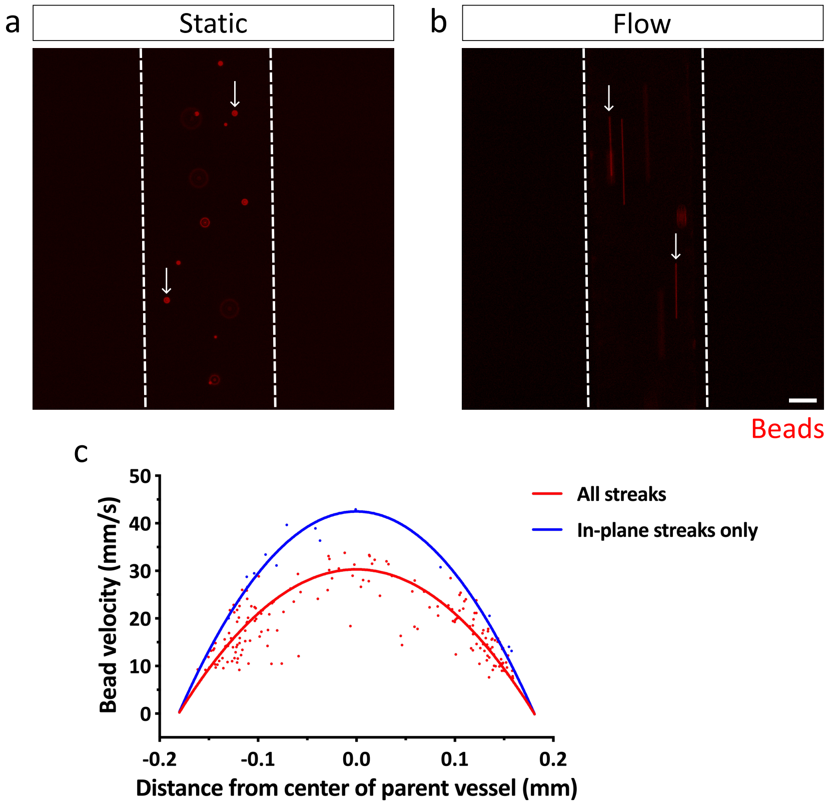

Fig. S5 Characterization of flow through the parent vessel. Fluorescent beads of 4 μm diameter (red) flowing through a parent vessel without (a) and with (b) flow. Scale bar: 100 μm. (c) Bead velocity under flow was calculated based on streak length of fluorescent beads in the perfusion media imaged with an exposure time of 10 ms. Red data points indicate all streaks captured in the field of view, while blue points indicate streaks specifically at the midplane of the vessel. A quadratic regression (red and blue solid lines) was used to determine the flow rate (Q) and wall shear stress (t_w) (Table S1).

Flow parameters	All streaks	In-plane streaks only
R^2	0.599156	0.934355
Q (mm^3/s)	1.554975	2.179476
T_w (dyn/cm^2)	3.354399	**4.701576**

Table S1 Calculation of flow rate and wall shear stress from the bead velocity profile. The overall shape fit for velocity profile (Fig. S5c) is a parabolic curve, but there were several streaks shorter than would be expected with Poiseuille flow, resulting in a curve with a low R^2 value of 0.599 and an estimated shear stress of 3.354 dyn/cm^2, which is lower than expected. Beads that were slower than 90% of the maximum velocity in a given bin for position within the parent vessel, were assumed to be outside of the midplane. Limiting the analysis to midplane beads only resulted in a more parabolic velocity profile with an R^2 of 0.934, and a shear of 4.702 dyn/cm^2, much closer to the target of 5 dyn/cm^2.

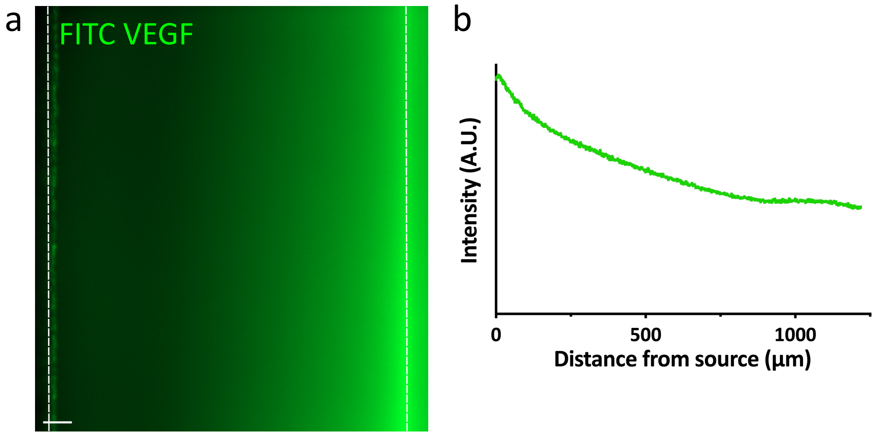

Fig. S6 Maintenance of FITC- conjugated VEGF-A gradient in DexVS hydrogel over 24 h in the presence of luminal flow. (a) VEGF-A (green) was added to the chemokine source channel and allowed to diffuse through the DexVS hydrogel towards the chemokine sink (parent) vessel under constant flow. Image was taken after 24 h. (b) Quantification of gradient formation of VEGF at 24 h. Scale bar: 100 µm.

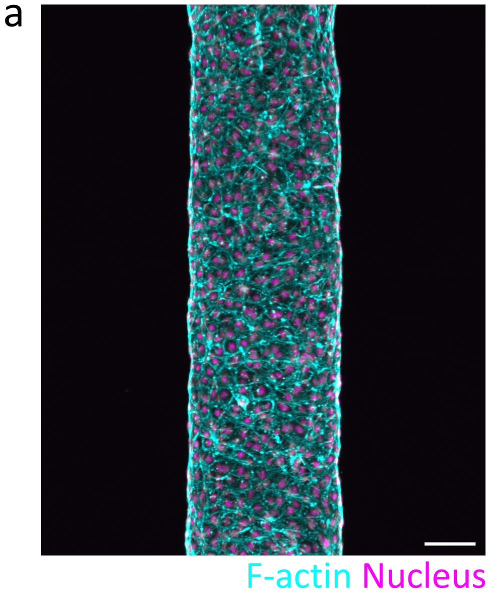

Fig. S7 Shear stress alone does not induce sprouting. (a) ECs exposed to high shear stress (5 dyn/cm^2) for 72 h do not sprout in the absence of a chemokine gradient. Scale bar: 100 µm.

a

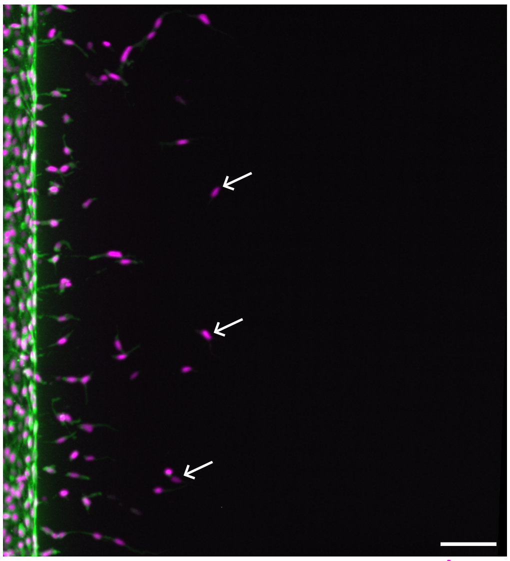

Fig. S8 ECs emanating from the parent vessel exposed to high shear stress (5 dyn/cm^2) lose Piezo1 expression as they invade further into the DexVS hydrogel. (a) ECs close to the parent vessel show elevated expression of Piezo1 (green), which they lose at further invasion distances. Scale bar: 100 µm.

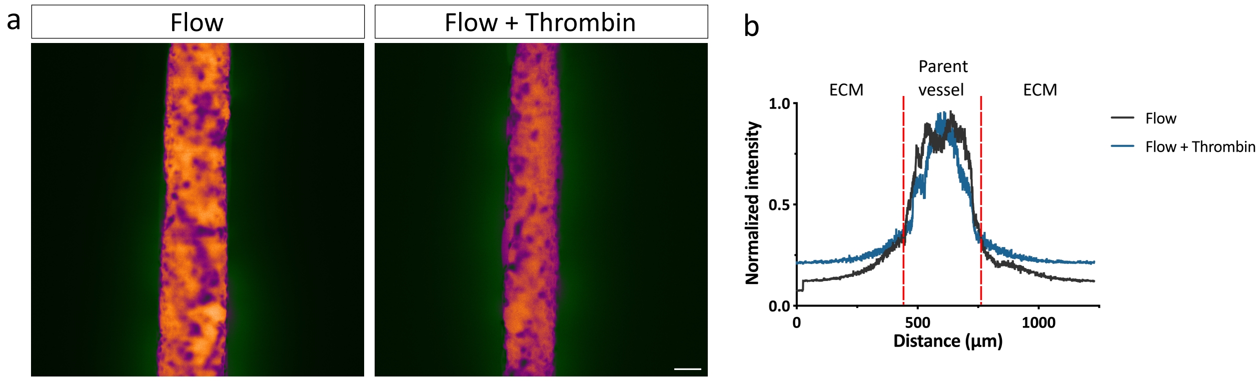

Fig. S9 Treatment with thrombin increases parent vessel permeability. (a) Representative images of parent vessel exposed to flow (5 dyn/cm^2) without (left) and with (right) treatment of thrombin, 10 min after FITC-dextran injection. Scale bar: 100 µm. (b) Quantification of intensity of FITC-dextran in the surrounding hydrogel, 10 min after its injection.

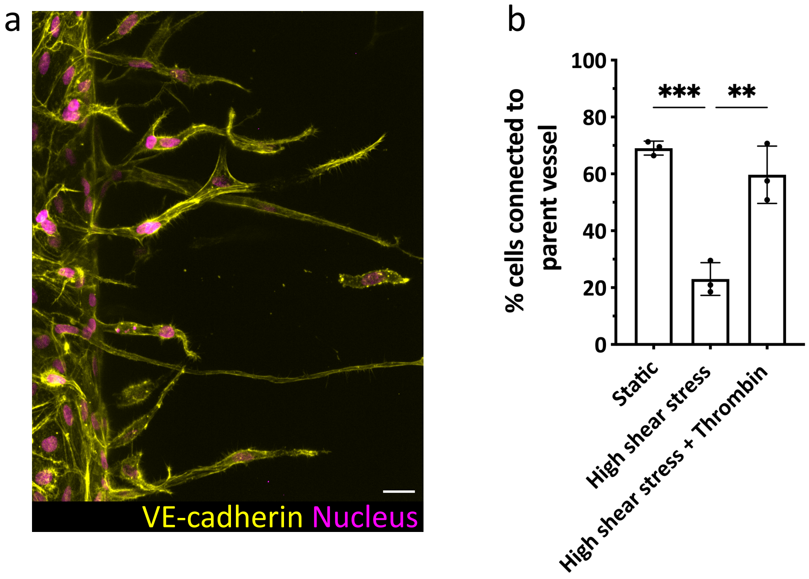

Fig. S10 Treatment with thrombin rescues connectivity of sprouts to parent vessel under flow (5 dyn/cm^2). (a) ECs in sprouts form VE-cadherin-positive junctions to ECs in parent vessel in the presence of high shear stress, when samples are treated with thrombin. Scale bar: 20 µm. (b) Quantification of percentage cells connected to the parent vessel relative to the total number of invaded cells in the hydrogel (in samples fixed at similar invasion depth). n = 3 experiments per condition. All data are presented as mean ± s.d., statistical significance was determined from $p < 0.05$ (Ordinary one-way ANOVA with Tukey's multiple comparisons test).
